# A Simple High-Resolution Near-Field Probe for Microwave Non-Destructive Test and Imaging

**DOI:** 10.3390/s20092670

**Published:** 2020-05-07

**Authors:** Zipeng Xie, Yongjie Li, Liguo Sun, Wentao Wu, Rui Cao, Xiaohui Tao

**Affiliations:** 1 School of Information Science and Technology, University of Science and Technology of China, Hefei 230027, China; xzp1993@mail.ustc.edu.cn (Z.X.); lyj16@mail.ustc.edu.cn (Y.L.); wwt@ustc.edu.cn (W.W.); 2 East China Research Institute of Electronic Engineering, Hefei 230088, China; cetc38caorui@126.com (R.C.); leotau@163.com (X.T.)

**Keywords:** complementary spiral resonators (CSRs), high resolution, microwave near-field imaging, non-destructive test

## Abstract

Non-destructive tests working at lower microwave frequencies have large advantages of dielectric material penetrability, lower equipment cost, and lower implementation complexity. However, the resolution will become worse as the work frequencies become lower. Relying on designing the structure of high field confinement, this study realizes a simple complementary spiral resonators (CSRs)-based near-field probe for microwave non-destructive testing (NDT) and imaging around 390 MHz (λ = 769 mm) whereby very high resolution (λ/308, 2.5 mm) is achieved. By applying an ingenious structure where a short microstrip is connected to a microstrip ring to feed the CSR, the probe, that is a single-port microwave planar circuit, does not need any extra matching circuits, which has more application potential in sensor arraying compared with other microwave probes. The variation of the electric field distribution with the standoff distance (SOD) between the material under test and the probe are analyzed to reveal the operation mechanisms behind the improved sensitivity and resolution of the proposed probe. Besides, the detection abilities of the tiny defects in metal and non-metal materials are demonstrated by the related experiments. The smallest detectable crack and via in the non-metal materials and the metal materials are of a λ/1538 (0.5 mm) width, a λ/513 (1.5 mm) diameter, a λ/3846 (0.2 mm) width and a λ/513 (1.5 mm) diameter, respectively. Moreover, to further evaluate the performance of the proposed probe, the defects under skin layer in the multilayer composite materials and the defects under corrosion in the carbon steel are inspected and imaged. Due to lower work frequency, high resolution, outstanding detection abilities of tiny defects, and large potentials in sensor arraying, the proposed probe would be a good candidate for microwave NDT and imaging.

## 1. Introduction

Microwave near-field imaging techniques are widely applied in non-destructive testing (NDT) of materials, due to their unique characteristics, such as no need for a coupling agent, contactless operation, dielectric penetrability, and low implementation costs [1]. Microwave imaging can be primarily categorized into two types: non-resonant type and resonant type [2,3]. Non-resonant microwave imaging generally uses open-ended waveguides as probes [4,5,6,7,8,9,10,11] and has been successfully implemented to detect cracks and corrosion on metal surfaces [4,5,6]. Additionally, microwaves could sense the permittivity variations caused by abnormalities in the materials. Therefore, the open-ended rectangle waveguide is implemented in defect inspection of composite materials and imaging of biological samples [7,8,9,10,11]. It is found that the sidelobes of open-ended circle waveguides are lower than those of rectangular ones, therefore, they are proved to have higher resolution in microwave imaging [12,13]. Nevertheless, the resolution of open-ended waveguides is limited by their apertures, and smaller apertures mean higher working frequencies which would weaken the ability of penetrating into the materials under test, and bring high equipment costs and implementation complexity [2].

Regarding the microwave imaging of the resonant type, open-ended waveguides loaded with resonators are proposed to improve the inspection performances [14,15,16,17,18], such as dielectric resonators and dipole antennas, which load open-ended waveguides to strongly enhance near field and improve their sensitivity and resolution [14,15,16,17]. The key of improving resolution is to concentrate electric fields into a small area. Electrically small resonators with high confinement of electromagnetic fields could create extremely concentrated electromagnetic fields and would be good microwave probe candidates. Split-ring resonators (SRRs), spiral resonators (SRs) and their complementary structures (CSRRs, CSRs) are kinds of electrically small resonators, which are known as metamaterial particles. SRRs excited by the open-ended waveguides could enhance the near field of the aperture and improve the probe resolution [18,19].

Actually, electrically small resonators could better excited by planar circuits. For SRRs, they usually excited by being coupled with a microstrip line. For CSRRs and CSRs, they are suitable to being etched in the ground of a microstrip, and they would strongly excited by the surface currents of the ground. Due to the strong near field of these electrically small resonators, they are applied for materials permittivity characterization [20,21,22,23], blood glucose tests [24], and materials surface and subsurface NDT and imaging [1,3,25,26,27,28,29,30] with high sensitivities and high resolutions. The above sensors based on electrically small resonators and excited by planar circuits are two-port structures. The resonant properties would appear in the transmission spectra as transmission zeroes. Generally, single-port probes are simpler to implement than two-port probes, and they are easier to form in a probe array. The single-port structure is also studied. The single-port sensors based on CSRRs are researched in materials permittivity characterization [31] and crack detection on metal surfaces [32]. SRs and CSRs are more compact than SRRs and CSRRs, therefore, it is easier to realize probes working at lower frequencies. A simpler single-port probe based on SRs has been presented. A loop antenna around SRs is needed to excite it, and a necessary matching circuit is used to match the probe to the 50 Ω coaxial feed line. This probe could achieve 5 mm (λ/140) spatial resolution at 433 MHz in defect imaging of composite materials, but its resolution is limited to the separation of the electric field peaks [2].

In this paper, we propose a much simpler single-port compact probe working around 390 MHz (λ = 769 mm) for microwave NDT and imaging. In order to overcome the resolution limitation of low frequency operation, after field confinement analysis, methods of avoiding the electric field dip around the center of the CSR and designing compact CSRs with a higher near-field confinement are adopted to improve the resolution. A high resolution (λ/308, 2.5 mm) is achieved. An ingenious structure, a microstrip ring with a short 50 Ω microstrip, is utilized to feed the CSR to make the proposed probe a compact microwave single-port planar circuit. It does not need an extra matching circuit, which makes it very easy to form a probe array in a microwave single-layer board. To evaluate the performance of the proposed probe, the very tiny defects of metal and non-metal materials are inspected and imaged. Additionally, the abilities of the proposed probe for detecting defects under a skin layer and corrosion are demonstrated.

## 2. Probe Design

### 2.1. Fields Confinement Analysis of CSRs

**Figure 1:**
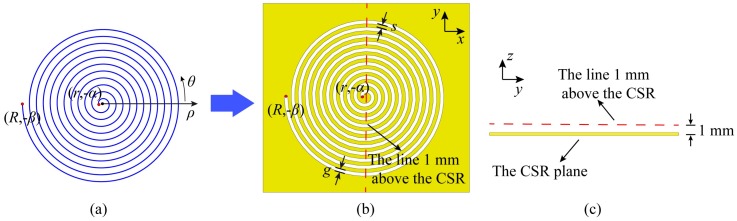
(**a**) The spiral in the polar coordinate; (**b**) The dimensions of CSR and the red dashed line 1 mm above the CSR; (**c**) The side view of the CSR and the red dashed line.

Etching a slot in the metal flat along a spiral could generate a CSR. The spiral defined in the polar coordinate and used to generate the CSR is shown in Figure 1a, where (*r*, −*α*) and (*R*, −*β*) are the endpoints of the spiral in the polar coordinate. The dimensions of the CSR are illustrated in Figure 1b,c. The spiral equation is shown in Equation (1). The parameters determining the CSR are *g*, *s*, *α*, *β*. *g* is the width of the spiral slot, and *s* is the width of the spiral metal strip. *R* is the outer radius of the spiral and *r* is the inner radius. *α* and *β* are the start angle and the end angle, respectively.

(1) $$\begin{array}{l} \rho =-\frac{1}{2\pi }\left(g+s\right)\theta \\ r\leq \rho \leq R\\ \alpha \leq -\theta \leq \beta \end{array}$$

**Figure 2:**
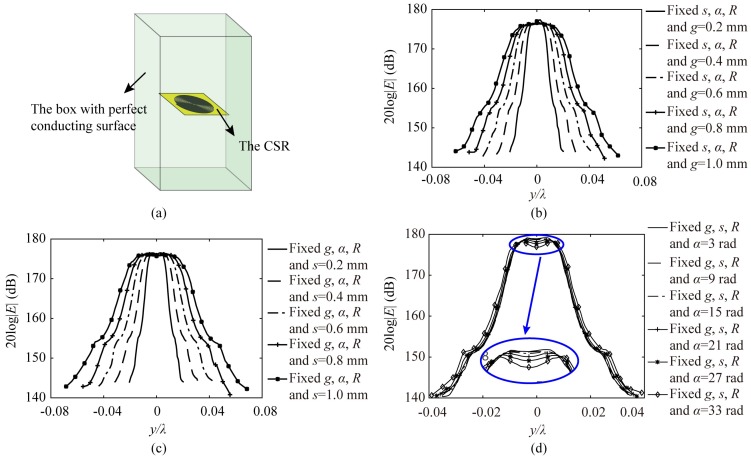
(**a**) The sketch of CSR set into the box of perfect conducting surfaces for eigenmode analysis; (**b**) The electric fields distributions along the red dashed line 1 mm above the CSRs varying with the width of spiral slots *g*; (**c**) The electric fields distributions along the red dashed line 1 mm above the CSRs varying with the width of the spiral metal strip *s*; (**d**) The electric fields distributions along the red dashed line 1 mm above the CSRs varying with the start angle of the spiral *α*.

The numerical analysis is an important and valid method for engineering study [1,2,3,12,13,14,15,16,17,18,19,20,21,22,23,24,25,26,27,28,29,30,31,32,33,34]. The eigenmode numerical analysis used to analysis the field confinement of CSRs is a simple and accurate way to analyse resonant structures, in which the equations of the free space wavenumber, the field, and mesh parameter are solved to get the resonance frequency and the distribution of electric fields. In this work, the eigenmode solver of the High Frequency Structure Simulator (HFSS) is applied for simulation [35]. The details of eigenmode numerical analysis could be found in the help documents of HFSS. In the HFSS software, the CSR is set into a box of appropriate size to make a mixture structure as shown in Figure 2a. The surfaces of the box are perfect electric conductors, and inside of it is vacuum. All the eigenmodes of the mixture structure are calculated. Hence, we need identify which eigenmodes are belonging to the CSR according to their field distributions.

The relationship between the field distribution and the structure parameters of CSRs is analyzed, and all the field distributions are calculated at the resonance frequencies of the CSRs. We keep the total dimension (*R*) constant (*R* = 6 mm), and analyze field distribution with the variations of *g* and *s* along the red dashed line which is 1 mm above the CSR plane as shown in Figure 1b,c. The results are illustrated in Figure 2b,c. It is observed that when *g* and *s* get smaller, the confinement of electric fields get higher. Furthermore, we also change the start angle *α*, while keep the total dimension (R) constant, and the results of field distribution are plotted in Figure 2d. It could be observed that the influence of the start angle *α* variation is not as big as that of *g* and *s*. When the start angle is increased, the confinement of electric fields gets worse, and the distribution of electric fields would get a dip caused by fields decay in the center of it. Two electric field peaks appear, which could limit the resolution of CSRs based probe [2]. Summarily, the analyses above would provide a guidance for the design of the CSR based probe that the smaller width of spiral slots (*g*), the smaller width of spiral metal strips (*s*) and the smaller start angle (*α*) would make a higher-resolution CSRs-based probe.

### 2.2. The CSRs-Based Probe

**Figure 3:**
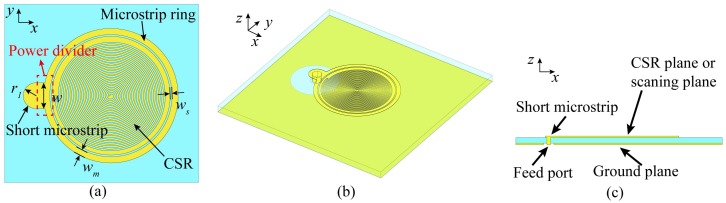
The sketch of the proposed probe: (**a**) the top view; (**b**) the three-dimensional view; (**c**) the cutaway view.

A very compact CSR is proposed, and excited by a simple microstrip ring with a short microstrip, as illustrated in Figure 3a–c. It is a simple single-port structure manufactured on a Rogers 4350 B (P.C.B.A Electronic (WuXi) Ltd., Wuxi, China) substrate with a thickness of 0.762 mm, and a relative permittivity of 3.66. According to the conclusions of Section 2.1, the parameters *s*, *g*, and *α* should set as small as possible to get a high near-field confinement and guarantee a high resolution of the CSRs-based probe. Because of the limitation of the printed circuit board technology, these parameters are set as *s* = 0.11 mm, *g* = 0.11 mm, *α* = 1 rad. The end angle of the spiral is set as *β* = 33π rad, which would provide a low resonance frequency for the CSR. The total dimension of the spiral *R* is 3.63 mm. The CSR is surrounded by a microstrip ring with a short microstrip. The characteristic impedance of the short microstrip is 50 Ω, and its dimensions are *w* = 1.7 mm, *r_1_* = 0.85 mm. The connection part between microstrip ring and the short microstrip is actually a power divider, as shown in Figure 3a, therefore, the characteristic impedance of microstrip ring is 100 Ω, and its width is *w_m_* = 0.4 mm. The width of the slots between the microstrip ring and the CSR are optimized by simulation, and it is *w_s_* = 0.12 mm. The short microstrip is connected to a 50 Ω coaxal port directly through a via as illustrated in Figure 3c. The proposed probe doesn’t need extra matching circuits since the power could be transmitted to the microstrip ring and coupled to the CSR with very little reflection.

**Figure 4:**
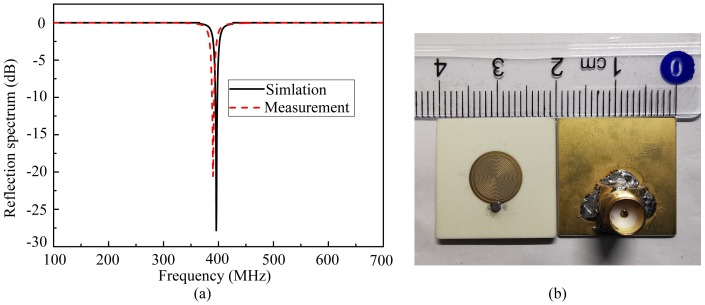
(**a**) The simulated and measured results of the proposed probe without materials under test. (**b**) The picture of the proposed probe.

By the numerical analysis, the reflection coefficient of the proposed probe without materials under test (MUT) could be gained. As shown in Figure 4a, the probe resonates at 396 MHz, and the minimum reflection coefficient is −28 dB. Additionally, the probe has been fabricated by the printed circuit board technology as shown in Figure 4b. A 50 Ω subminiature version A (SMA) connector, welded to the short 50 Ω microstrip by drilling a via, is linked to a vector network analyzer (VNA) through a 50 Ω coaxial line to measure the reflection coefficient. The measured result is also illustrated in Figure 4a. From the Figure 4a, it is observed that the minimum reflection coefficient is −22 dB, and the resonance frequency is 390 MHz, which is shifted slightly compared with the simulation. It may be caused by fabrication deviations. Additionally, the probe is plated with gold. Since the conductor loss of gold is higher than that of copper, which could lead to the increase of the minimum reflection coefficient. Generally, the measured results agree with the simulation and the numerical analysis is validated.

## 3. Sensitivity and Resolution Evaluation

**Figure 5:**
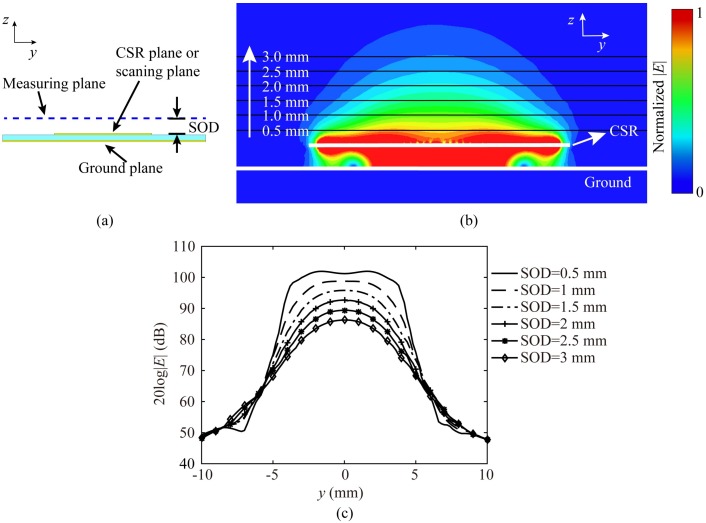
(**a**) The sketch to show the measuring plane, the CSR plane (or scanning plane) and SOD; (**b**) The electric field distribution of the probe in the plane yoz; (**c**) The electric fields distributions varying with SOD along y-axis.

It is noticed that it is the near field that the probe relies on to image MUT, rather than the far radiation field. The radiation efficiency of the probe is calculated at the resonance frequency by numerical analysis, and the result is 2.32 × 10^−6^, which means the radiation energy of the proposed probe is extremely small. The loss of metal and dielectric is also very small at low frequencies, therefore, most of the electromagnetic energy is confined in the near region of the probe surface. The distance between the measuring plane and the scanning plane is called the standoff distance (SOD) as shown in Figure 5a. 

The electric field distribution in the profile of the probe has been obtained by numerical analysis, and it is plotted in Figure 5b. It reveals the fact that the intensity of electric fields decay rapidly as they get away from the CSR plane which is also called the scanning plane. It is observed that the localizations of electric fields are different at the measuring planes with different SODs. The electric field distributions of different SODs along y-axis are shown in Figure 5c.

The localization of electric fields could be described by the half electric power beamwidth (HEPB). The HEPB has directly influences on the resolution of the probe. Therefore, we list the HEPBs of different SODs at Table 1. The HEPB first decrease and then increase with the increase of the SOD, and it gets the smallest value when the SOD is 2.5 mm. The variation of HEPB could be used to predict the variation of resolution as the SOD changes.

**Figure 6:**
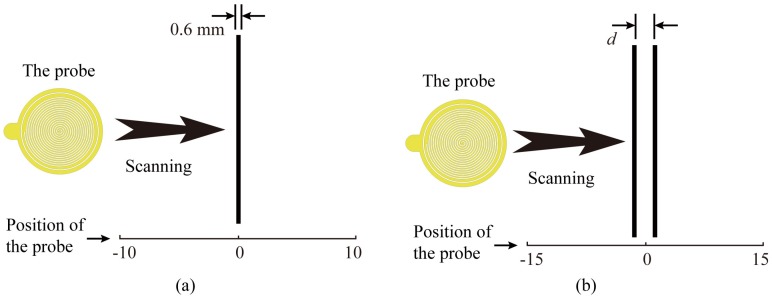
(**a**) The sketch of the sensitivity evaluation by the probe scanning a conducting wire; (**b**) The sketch of the resolution evaluation by probe scanning two adjacent conducting wires.

The sensitivity and resolution are the most important factors. Generally, these two factors are different when imaging different targets. For comparison, generally, the sensitivity evaluation is accomplished by scanning a conducting wire with a distance of SOD, as shown in Figure 6a. And the resolution is obtained by scanning two same adjacent conducting wires with a distance of SOD as shown in Figure 6b.

**Figure 7:**
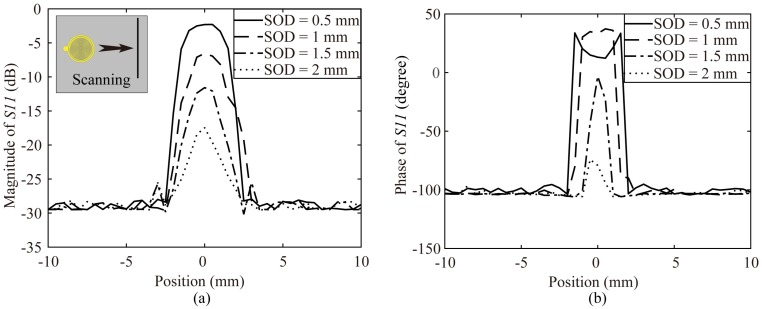
(**a**) The simulated magnitude variation of the reflection coefficient for the proposed probe scanning a conducting wire with different SODs; (**b**) The simulated phase variation of the reflection coefficient for the proposed probe scanning a conducting wire with different SODs.

As mentioned above, the SOD is an important factor for the measurement process. The intensity of the electric fields decays quickly, as the measuring plane gets away from the CSR plane. We could predict that the sensitivity would be worse while the SOD is increased. We utilize numerical analysis to study how the SOD affects the sensitivity by the probe scanning a 0.6 mm diameter conducting wire with different SODs as shown in Figure 6a. The results for the magnitude and phase variation of the reflection coefficient are illustrated in Figure 7a,b. It is observed that the maximum variations of both magnitude and phase increase as SODs get smaller, which imply that the sensitivity get better as SODs reduced. It is worth noting that when the SOD is too small, the phase variation would be saturated, as presented in Figure 7b. Additionally, when the SOD is set too big, the maximum variations of magnitude and phase would be rapidly reduced.

**Figure 8:**
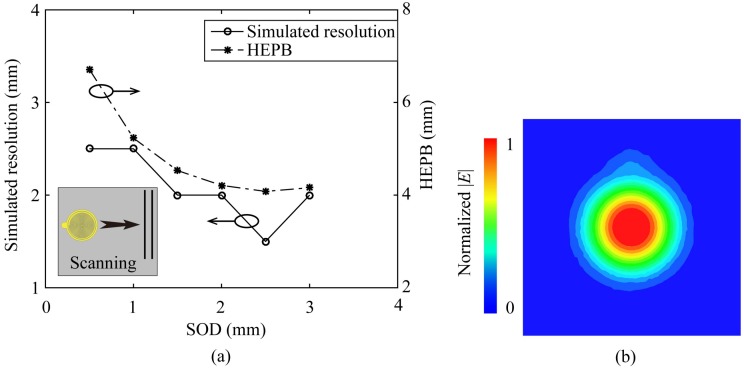
(**a**) The HEPB variation and the simulated resolution evaluation for different SODs, and the simulated resolution is obtained by scanning two adjacent conducting wires; (**b**) The electric near field distribution in the measuring plane of a 1 mm SOD.

For the resolution evaluation, as illustrated in Figure 6b, two same adjacent 0.6 mm diameter conducting wires are set under the probe with a SOD, and probe scans the wires along the perpendicular direction of the wires. The distance between two wires is *d*. The probe scans two adjacent wires and the distance *d* is decreased from a big value like 4 mm to 1 mm in steps of 0.5 mm. When the probe could just distinguish the two wires and any more decreases of the distance *d* would cause the two wires indistinguishable, the smallest distance *d* is defined as the resolution of the probe. The resolutions obtained by scanning two same adjacent conducting wires (0.6 mm diameter) with different SODs are plotted in Figure 8a, and the HEPBs of the corresponding SODs are also plotted as comparison. The resolution gained by scanning two same conducting wires is an approximate value, because the resolution is ascertained by a 0.5 mm variation of the distance *d* between two wires. There is the best SOD making the resolution highest, and it is 2.5 mm as agreed with the HEPB analysis. Generally, we could deduce that both of the HEPB and the resolution obtained by scanning two conducting wires could reflect the spatial resolution of the probe. They are approximately equivalent.

As discussed above, the sensitivity and the resolution are opposite when the SOD is set below 2.5 mm. Therefore, to make a compromise between the sensitivity and the resolution, and considering the practical operation, we set SOD as 1 mm in the imaging processing, of which the resolution is 2.5 mm (λ/308). The electric fields distribution in the measuring plane of a 1 mm SOD is plot in the Figure 8b. It is observed that the electric near fields form a spot above the center of the CSR, and there is no dip in the field distribution pattern as talked above, which would be beneficial to the resolution improvement.

**Figure 9:**
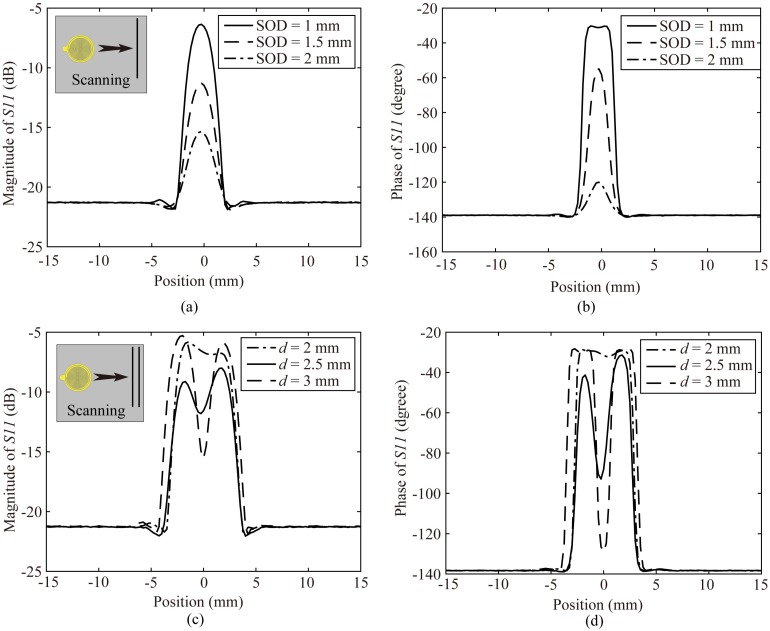
(**a**) The measured magnitude variation of the reflection coefficient for the proposed probe scanning a conducting wire with different SODs; (**b**) The measured phase variation of the reflection coefficient for the proposed probe scanning a conducting wire with different SODs; (**c**) The measured magnitude variation of the reflection coefficient for the proposed probe scanning two conducting wires separated by a distance *d* with an 1 mm SOD; (**d**) The measured phase variation of the reflection coefficient for the proposed probe scanning two conducting wires separated by a distance *d* with an 1 mm SOD.

To verify the numerical analysis, the related experiments have been done. One conducting wire is under scanning by the proposed probe with different SODs, and the measured results for the variation in magnitude and phase of the reflection coefficient are illustrated in Figure 9a,b. The measured results verify the verdict of numerical analysis that the sensitivity get better as SOD decreased. Whereby the proposed probe scans two conducting wire separated by a distance *d*, and the measured results of resolution are obtained. The measured results are demonstrated in Figure 9c,d. The measured results testify that the resolution of the proposed probe is 2.5 mm (λ/308) for a 1 mm SOD.

## 4. The Abilities of Conventional Defects Detection

### 4.1. The Cracks and Vias of Non-Metal Materials

**Figure 10:**
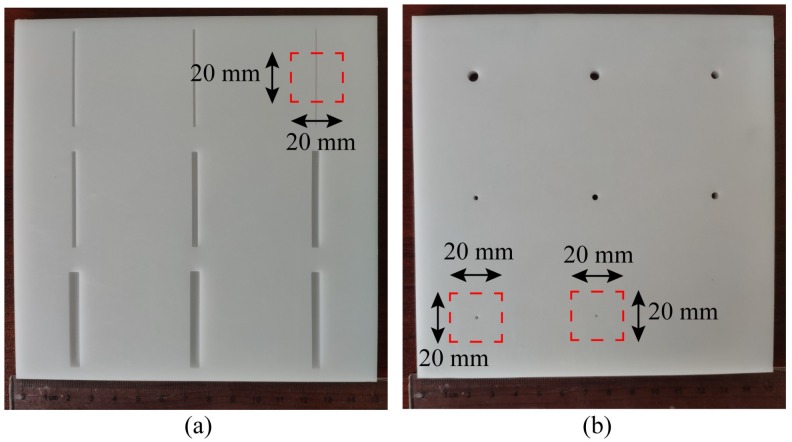
The two POM boards with different defects, and the regions of red rectangles are to be imaged: (**a**) The POM board with the cracks of different widths; (**b**) The POM board with the vias of different diameters.

The cracks and voids of non-metal materials are very common defects. The detection abilities of these small defects are quite significant in the non-destructive test of non-metal materials. However, machining the voids in materials is difficult, therefore, voids are replaced by vias in our situation. We machine two polyformaldehyde (POM) boards. One is etched with the cracks of different widths, and the other is drilled with the vias of different diameters, as shown in Figure 10. All these cracks and vias in the POM board are machined by computer numerical control lathe. The widths of cracks are 0.5, 1, 1.5, 2, 2.5, 3, 3.5, 4, and 4.5 mm and the depths of all cracks are 2 mm. The diameters of these vias are 0.5, 1, 1.5, 2, 2.5, 3, 3.5, 4 and 4.5 mm.

**Figure 11:**
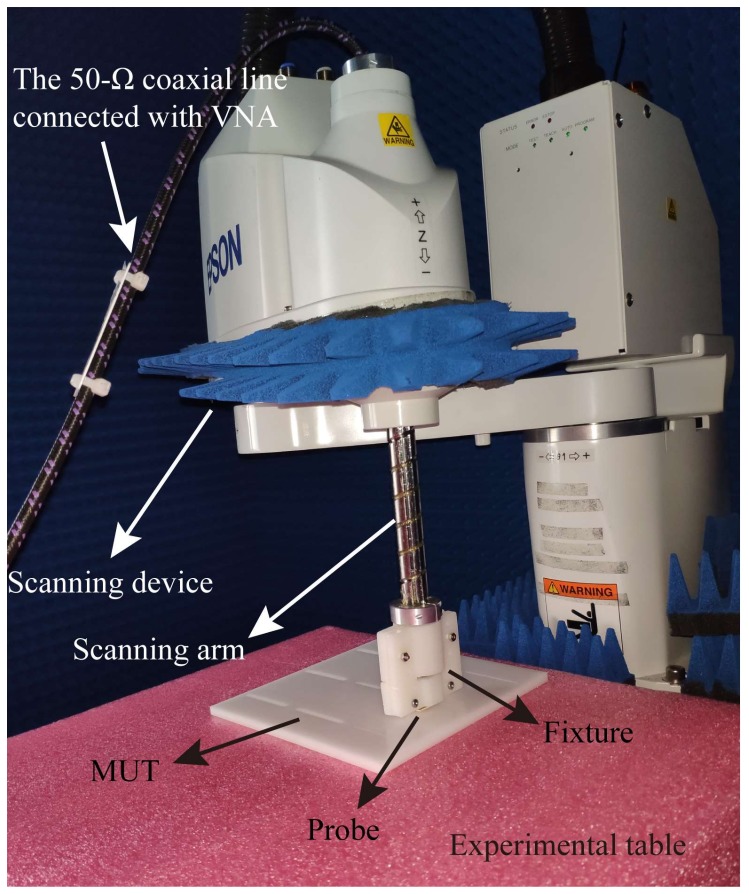
The testing environment.

The testing environment is demonstrated in Figure 11. The proposed probe is fixed on the scanning arm of the scanning device by the fixture. A 50 Ω coaxial line is applied to transmit the signal from the probe to VNA. One end of the line is connected to the probe, and another end is connected to VNA. The POM board is set on the experimental table. VNA and the scanning device are both connected to computer for controlling. The scanning parameters setting, including the size of scanning area, the scanning step, the measuring frequencies, and the storage path of the result file, is done in computer. After setting the scanning parameters, the scanning and the measurement will proceed automatically.

**Figure 12:**
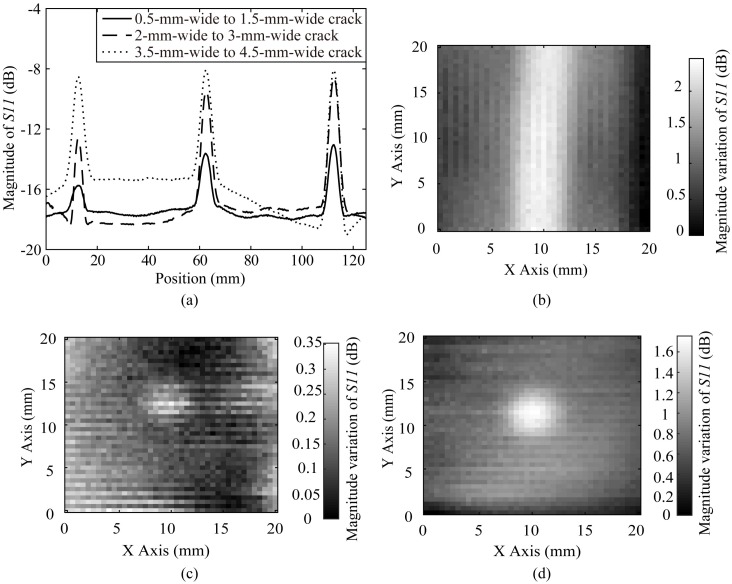
(**a**) The magnitude responses for the probe scanning the cracks in the POM board of different widths; (**b**) The image of the 0.5 mm width crack obtained by the magnitude variation; (**c**) The image of the 1 mm diameter via obtained by the magnitude variation; (**d**) The image of the 1.5 mm diameter via obtained by the magnitude variation.

The proposed probe scans the POM boards with a 1 mm SOD. Whereby the proposed probe scans the cracks of different widths with a 0.5 mm step, the magnitude responses of the reflection coefficients for the probe are plotted in Figure 12a. It is verified that the proposed probe could sense a 0.5 mm wide and 2 mm deep crack. The magnitude variation of *S11*, which is called as the magnitude dynamic range, is increased as the widths of cracks grow. Additionally, the probe scans a 20 mm × 20 mm square area that contain the 0.5 mm wide crack as the region of the red rectangle, and the image is gained by calculating the magnitude variation of the reflection coefficient as illustrated in Figure 12b. The crack is very clear in Figure 12b in an around 2.5 dB dynamic range, which confirms that the proposed probe could detect the crack of a λ/1538 (0.5 mm) width. The 1 mm diameter and 1.5 mm diameter vias in the POM board have also been imaged by calculating the magnitude variation of the reflection coefficient, the results are plotted in Figure 12c,d, respectively. The image for the via of 1 mm diameter is indistinct as demonstrated in Figure 12c, while the via of 1.5 mm diameter is discernible in Figure 12d which is imaged in 1.6 dB dynamic range. Hence, the smallest detectable via is the 1.5 mm diameter via. It proves the fact that the proposed probe could inspect the via of a λ/513 (1.5 mm) diameter around 390 MHz (λ = 769 mm).

### 4.2. The Cracks and Vias of Metal Materials

**Figure 13:**
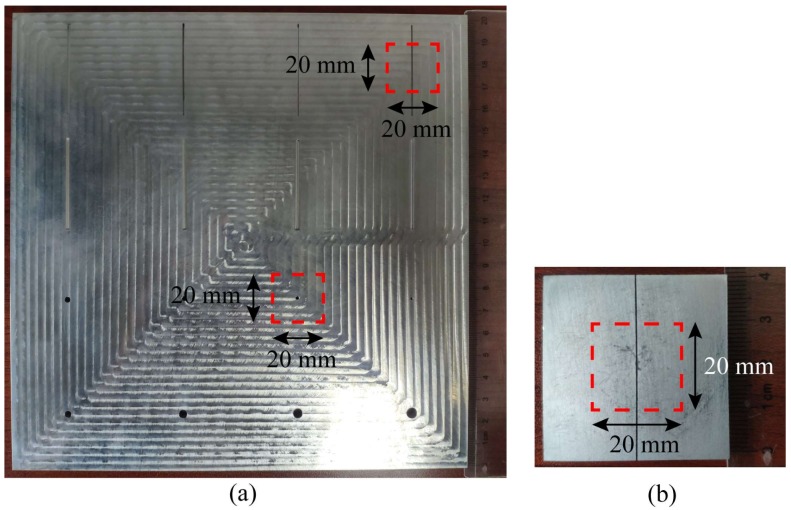
The pictures of metal boards, and the regions of red rectangles are to be imaged: (**a**) the aluminum board fabricated with cracks and vias; (**b**) the aluminum board etched with a 0.2 mm wide crack.

In metal materials, cracks and holes are also common defects. Detecting these defects is the most important objective of metal materials NDT. Therefore, the abilities of the probe for inspecting small cracks and holes are needed to be evaluated. Cracks of different widths and the vias of different diameters are machined in the aluminum board by a computer numerical control lathe, as shown in Figure 13a. The surface of aluminum board seems to be rugged. It is quite glossy in fact. The spoors of machining actually are very small although they are amplified by the reflected light in the picture. The widths of these cracks are 0.5 0.6, 0.8, 1.2, 1.4, 1.6, 1.8 and 2 mm, and the depths of all cracks are 2 mm. The diameters of these vias are 1, 1.5, 2, 2.5, 3, 3.5, 4 and 4.5 mm. For the tiny crack, we machine another aluminium board, and etch a 0.2 mm wide and 2 mm depth crack on it by the wire electrical discharge machining technology (WEDM technology), as shown in Figure 13b.

**Figure 14:**
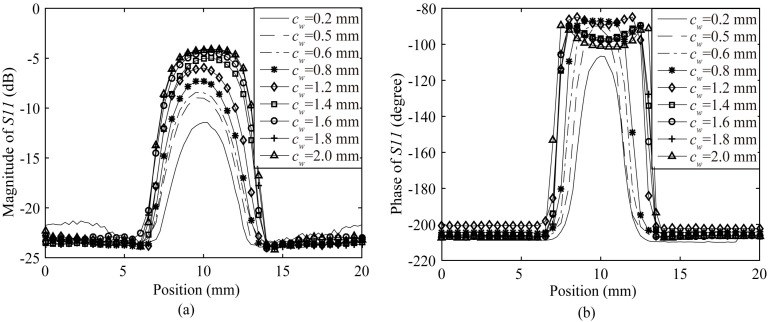
(**a**) The magnitude variations of the reflection coefficient for the probe scanning the cracks of different widths; (**b**) The phase variations of the reflection coefficient for the probe scanning the cracks of different widths.

The proposed probe scans the cracks of different widths by 1 mm SOD so as to estimate the performance of the proposed probe in cracks detection of metal materials. The scanning results of both magnitude and phase responses are illustrated in Figure 14a,b, respectively. In Figure 14a, as the widths of cracks (*c_w_*) increase, the magnitude dynamic ranges also increase. But the magnitude dynamic ranges would reach a saturation value when widths of cracks grow big enough. In Figure 14b, when the widths of cracks get bigger (not more than 0.8 mm), the phase dynamic ranges become larger. When the width of the crack is over 0.8 mm, a dip would appear in the middle of the phase variation curve. As the width of the crack continues to increase beyond 0.8 mm, the phase dynamic range does not grow any more, and the dip in the middle of the phase variation curve becomes deeper, which means the phase dynamic range is saturated. It could be observed from Figure 14a,b that the proposed probe could inspect a 0.2 mm wide crack.

**Figure 15:**
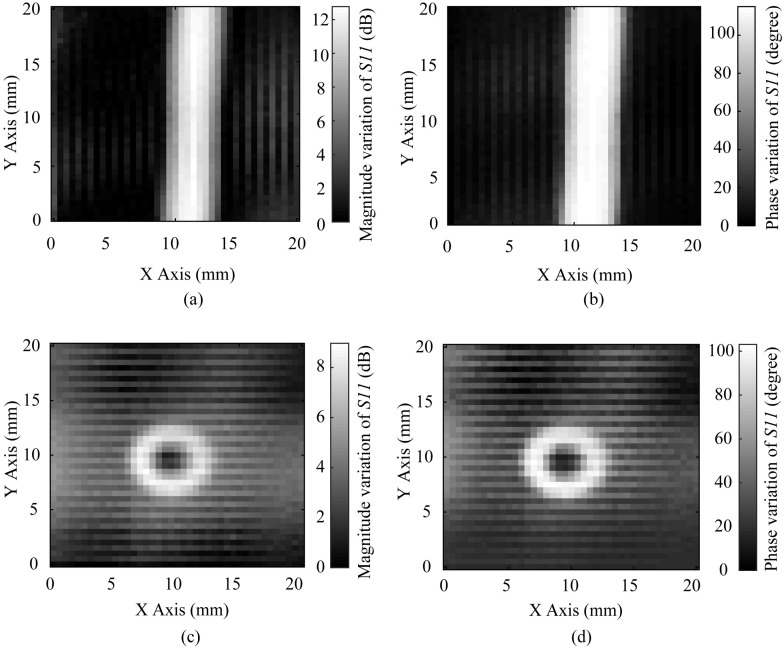
(**a**) The image of the 0.2 mm wide crack in the aluminum board obtained by the magnitude variation; (**b**) The image of the 0.2 mm wide crack obtained by the phase variation; (**c**) The image of the 1.5 mm diameter via obtained by the magnitude variation; (**d**) The image of the 1.5 mm diameter via obtained by the phase variation.

Furthermore, we image the 0.2 mm wide crack as illustrated in Figure 15a of a 12 dB magnitude dynamic range by calculating the magnitude variation of the reflection coefficient, and as illustrated in Figure 15b of a 100° phase dynamic range by calculating the phase variation of the reflection coefficient. The ability for the proposed probe of imaging a λ/3846-wide (0.2 mm wide) crack has been testified at 390 MHz. The vias in the aluminium board have also been detected, and the smallest detectable via in the aluminium board is the via of a λ/513 (1.5 mm) diameter. Its images are acquired by the magnitude variation and the phase variation, as shown in Figure 15c,d with an 8 dB magnitude dynamic range and 100° phase dynamic range, respectively. 

### 4.3. Two Samples for Further Evaluation of Imaging Abilities

**Figure 16:**
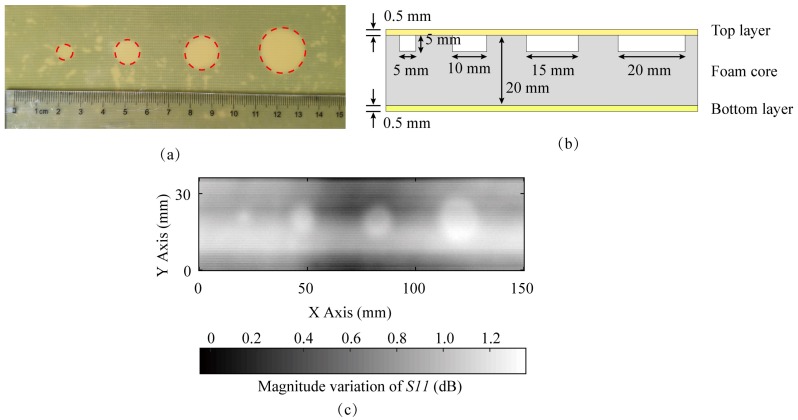
(**a**) The picture for sample 1 which is a multilayer composite board, and four holes of different diameters are drilled in its foam core; (**b**) the cutaway view of the sample 1; (**c**) the image gained by the magnitude variation.

Sample 1 is a multilayer composite board. The top layer and the bottom layer are fiberglass boards, with thicknesses of 0.5 mm. The middle layer is the polyurethane resin foam core, which is sandwiched by the fiberglass boards, and its thickness is 20 mm. Holes of different diameters have been drilled in the foam core, as shown in Figure 16a,b. The diameters of these holes are 5, 10, 15 and 20 mm and the depth of all holes is 5 mm. The sample has been set on the experimental table and scanned by the proposed probe. The magnitude variation is calculated to obtain the image result as shown in Figure 16c of the magnitude dynamic range around 1.2 dB. All holes could be observed in Figure 16c, and the abilities of the proposed probe for detecting the defects under dielectric materials are verified.

**Figure 17:**
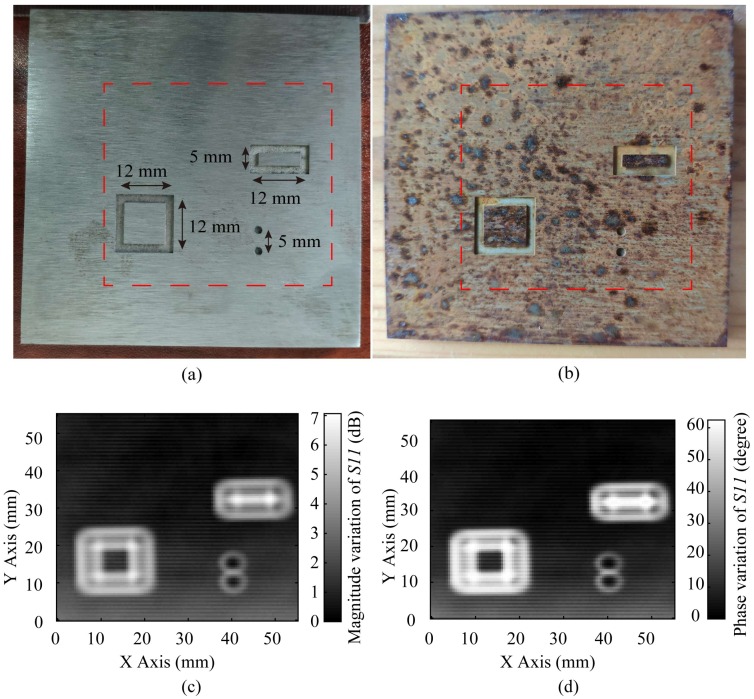
(**a**) The unrusted carbon steel board with patterns etched; (**b**) The rusted carbon steel board with patterns etched; (**c**) The image of the rusted carbon steel acquired by the variation of *S11* magnitude; (**d**) The image of the rusted carbon steel acquired by the variation of *S11* phase.

Sample 2 is a carbon steel board with four 0.5 mm deep patterns etched on it, and the dimensions of those patterns are illustrated in Figure 17a. Then, the carbon steel board is rusted, which is illustrated in Figure 17b. The obtained images by the proposed probe are demonstrated in Figure 17c with a 7 dB dynamic range in the magnitude variation of *S11* and Figure 17d with a 60° dynamic range in the phase variation of *S11*, which are very high dynamic ranges. 

In Figure 17c,d, the patterns in the rusted board can be clearly observed. It is proved that the defects under the rust could be detected by the proposed probe.

## 5. Conclusions

In this paper, we have designed a simple near-field microwave imaging probe with very high resolution (2.5 mm, λ/308) around 390 MHz. The simple planar structure without any extra matching circuits makes the probe well suited to sensor arraying. The proposed probe could detect and image a 0.5 mm width (λ/1538) crack, a 1.5 mm diameter (λ/513) via in non-metal materials, a 0.2 mm width (λ/3846) crack and a 1.5 mm diameter (λ/513) via in metal materials. Defects under a skin layer and corrosion also could be imaged by the proposed probe with a good dynamic range. In conclusion, its superior features such as high resolution, easy arraying, and good performance in tiny defects detection make the proposed probe have wide application prospects in microwave NDT and imaging. Nevertheless, the imaging efficiency of a single probe is not as good as that of an array, and an array of the proposed probe, that would improve the imaging efficiency, is not studied in this work. Studies on probe arraying will be done in the future.

## Figures and Tables

**Table 1:** HEPB varying with SOD.

Parameters	Values					
SOD (mm)	0.5	1	1.5	2	2.5	3
HEPB (mm)	6.71	5.23	4.53	4.21	4.08	4.16
